# Palliative end ileostomy and gastrojejunostomy for a metastatic distal transverse colonic malignancy complicated by a proximal duodenocolic fistula: a case report

**DOI:** 10.1186/s13256-017-1398-9

**Published:** 2017-08-14

**Authors:** Gnanaselvam Pamathy, Umesh Jayarajah, Yapa Hamillage Hemantha Gunathilaka, Sivasuriya Sivaganesh

**Affiliations:** 1 University Surgical Unit, National Hospital of Sri Lanka, Colombo, Sri Lanka; 2 Department of Surgery, Faculty of Medicine, University of Colombo, Kynsey Road, P.O. Box 271, Colombo 8, Western Province Sri Lanka

**Keywords:** Palliative end ileostomy, Malignant duodenocolic fistula

## Abstract

**Background:**

Fistulae between the colon and upper gastrointestinal tract are distressing and uncommon complications of malignancies involving this region. We report a case of a middle-aged man with a locally advanced and metastatic distal transverse colon malignancy who presented with a duodenocolic fistula proximal to the primary tumor and underwent palliative surgery.

**Case presentation:**

A 50-year-old Sri Lankan man presented to our hospital with a history of feculent vomiting of 1 week’s duration preceded by worsening constipation and abdominal fullness of 2 months’ duration. He also complained of anorexia and significant weight loss over the previous month. His physical examination was unremarkable except for his wasted appearance. Flexible sigmoidoscopy done at his local hospital had not revealed any abnormality in the left colon. Gastroduodenoscopy did not reveal fecal matter or any mucosal abnormalities in the stomach or duodenum. An abdominal contrast-enhanced computed tomographic scan showed a mid-to-distal transverse colonic tumor with a duodenocolic fistula proximal to the primary lesion. At laparotomy, he was found to have an unresectable, locally advanced mid transverse colon tumor with diffuse peritoneal and mesenteric deposits and mild ascites. Palliative end ileostomy and gastrojejunostomy were performed before closure. Histology from the malignant deposits revealed a well-differentiated adenocarcinoma. He made an uneventful recovery with good symptomatic relief.

**Conclusions:**

Malignant gastric or duodenocolic fistulae are uncommon complications of locally advanced colonic malignancies with direct invasion to the stomach or duodenum. Although the characteristic clinical presentation of feculent vomiting suggests the diagnosis, cross-sectional imaging is confirmative in addition to staging the disease. Management is guided by disease stage, nutritional status, and the general condition of the patient and ranges from extensive bowel resection including the fistula to palliative options.

## Background

Enteroenteric fistulae complicate malignancies and benign conditions such as Crohn’s disease, tuberculosis, actinomycosis, radiation enteritis, diverticulitis, and peptic ulcer disease, and they may rarely be iatrogenic [1–4]. Malignant enteroenteric fistulae more commonly occur between the ileum or jejunum and sigmoid colon malignancies [2]. Duodenocolic fistulae are rare complications of colonic malignancies, with a reported incidence of 0.14% [5]. Other rare causes include gallbladder carcinoma, duodenal adenocarcinoma and metastatic cancer of the esophagus [6]. Feculent vomiting and halitosis, which are characteristic of this condition, are extremely disturbing to the patient. Metabolic abnormalities and nutritional deficiencies are common sequelae of fistulae involving the upper gastrointestinal tract and the colon [2, 7]. The disease is often advanced at the time of diagnosis, and treatment is often palliative.

## Case presentation

A previously healthy 50-year-old Sri Lankan man presented to our hospital with feculent vomiting of 1 week’s duration. This was preceded by worsening constipation, abdominal fullness, and intermittent abdominal pain of 2 months’ duration. He denied passage of fresh or altered blood with stools. Interestingly, the abdominal discomfort and fullness and the sensation of a full rectum were at times relieved by feculent belching. He had significant weight loss over the previous month and associated anorexia. He was a nonsmoker and had not been on nonsteroidal anti-inflammatory drug therapy. He was dehydrated and appeared wasted, with a body mass index of 21 kg/m^2^. His general and abdominal examinations were otherwise unremarkable.

**Fig. 1:**
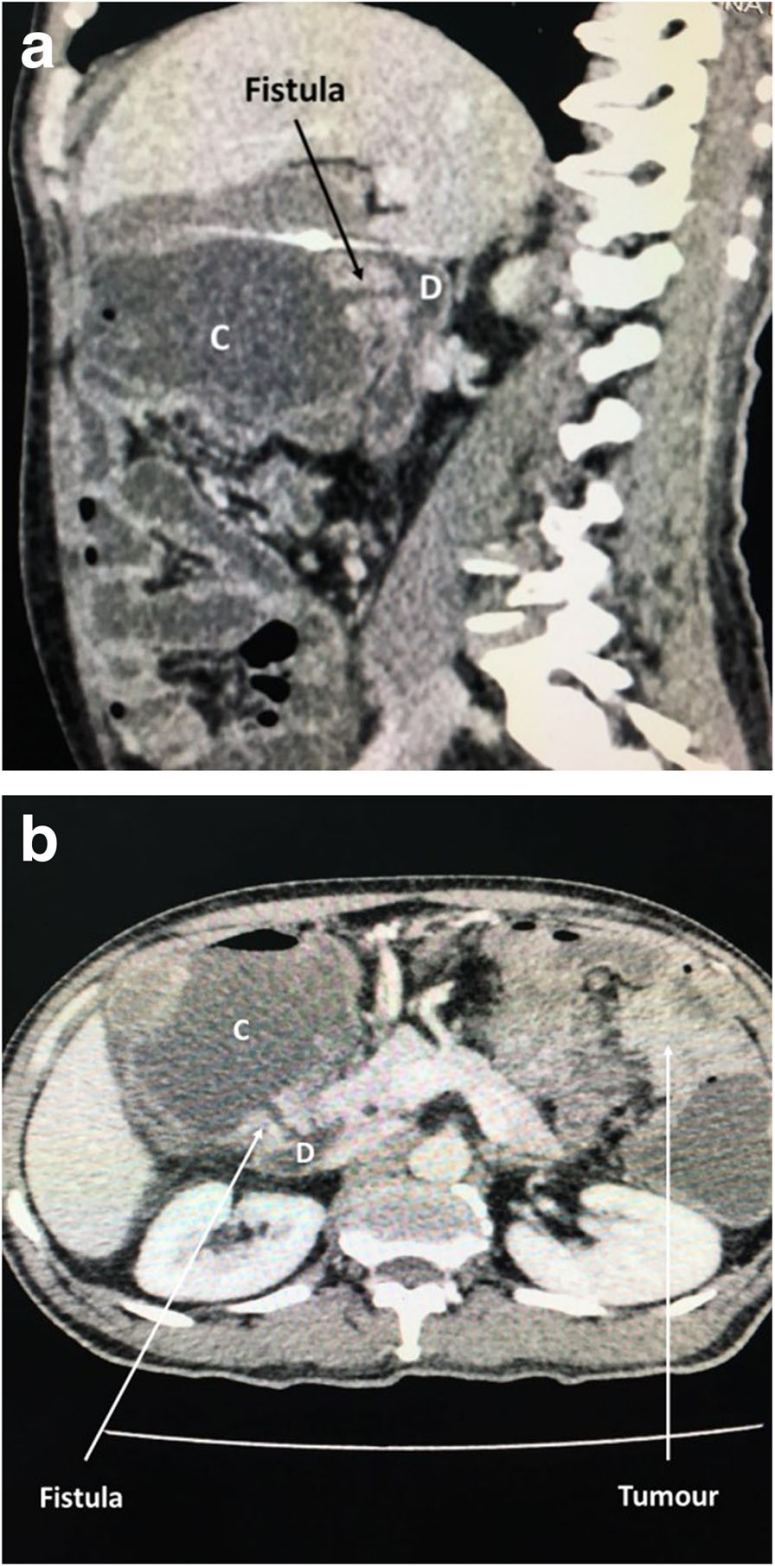
**a** Computed tomographic scan sagittal view demonstrating fistula between hepatic flexure (C) and duodenum (D). **b** Computed tomographic scan axial view demonstrating fistula between hepatic flexure (C) and duodenum (D) and distal transverse colon tumor

He had been investigated at his local hospital, where a flexible sigmoidoscopy had not revealed any abnormality in the left colon. Suspecting a malignant gastrocolic fistula, a gastroduodenoscopy was performed, but this did not reveal fecal matter or any mucosal abnormalities in the stomach or duodenum. An abdominal contrast-enhanced computed tomographic (CT) scan revealed a mid-to-distal transverse colonic tumor with a duodenocolic fistula proximal to the primary lesion (Fig. 1a and b). A colonoscopy was not performed, because the patient was generally unwell and was not amenable to drinking 3–4 L of bowel preparation, and also because of the possibility of acute intestinal obstruction and exacerbated feculent vomiting. Furthermore, on the basis of the CT findings, a colonoscopy was unlikely to provide new information that would alter the plan of management. The patient’s basic biochemistry was normal, but his carcinoembryonic antigen levels were elevated. He was scheduled for a laparotomy after a period of nutritional optimization including parenteral nutrition.

**Fig. 2:**
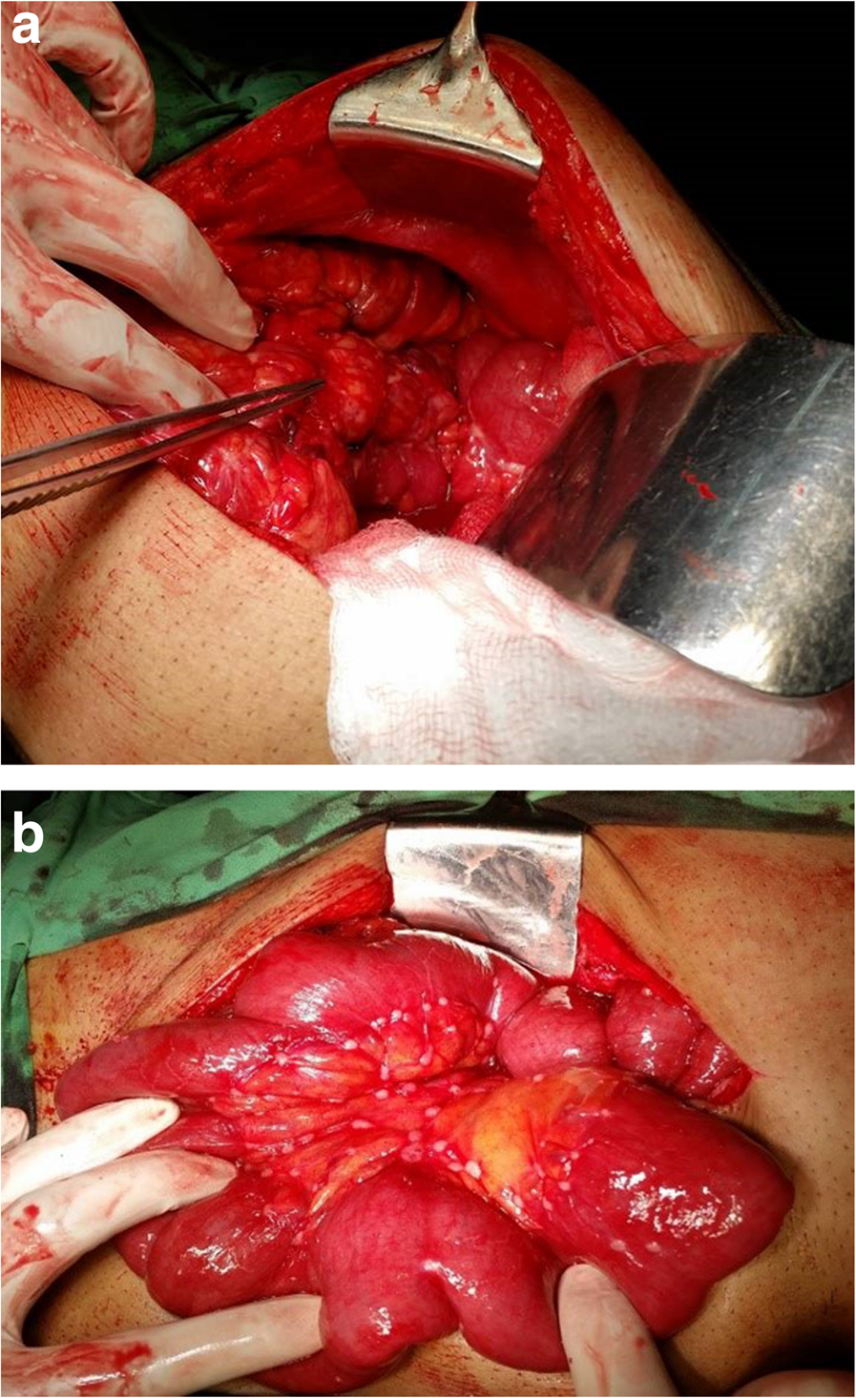
**a** Contracted distal transverse mesocolon with tumor infiltration **b** Multiple tumor deposits in small bowel mesentery

**Fig. 3:**
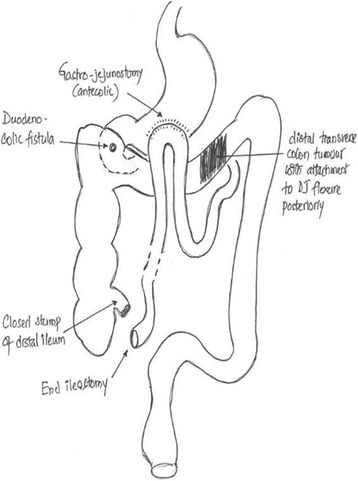
Diagram of palliative procedure performed. *DJ* duodenojejunal

At laparotomy, he was found to have an unresectable locally advanced mid-to-distal transverse colonic tumor with possible infiltration of the duodenojejunal (DJ) flexure. A small amount of ascites, diffuse peritoneal and mesenteric deposits with scirrhous contraction of the mesocolon and small bowel mesentery, was noted (Fig. 2a and b). An antecolic loop gastrojejunostomy was performed to bypass possible future obstruction at the DJ flexure. The terminal ileum was divided and brought out as an end ileostomy, thereby defunctioning the colon and effectively neutralizing the duodenocolic fistula (Fig. 3). Histology from the malignant deposits revealed a well-differentiated adenocarcinoma. He made a good recovery with relief of symptoms and was discharged on a normal diet 2 weeks after surgery. A postoperative oral contrast study showed good emptying of contrast via the gastroenterostomy and did not show the fistula.

Postoperatively, he received eight cycles of FOLFOX therapy (5-fluorouracil, folinic acid, and oxaliplatin), for which he showed an excellent response and was well and disease-free 10 months after surgery.

## Discussion

Duodenocolic fistulae are an uncommon form of gastrointestinal fistulae and a rare presentation of colonic malignancies around the hepatic flexure [1, 2]. As in this patient, they typically present with feculent vomiting but cannot be clinically distinguished from gastrocolic fistulae. They may cause electrolyte and metabolic abnormalities, nutritional deficiencies, and gastrointestinal bleeding [7]. The fistulae arise due to malignant erosion of a colonic tumor into the duodenum, stomach, or vice versa. Interestingly, in our patient, the tumor was in the mid-transverse colon, distal to the site of fistulation. The entire transverse mesocolon was grossly contracted and scirrhous, resulting in the normally redundant and mobile transverse colon being plastered to posterior structures. It is likely that the combination of an eroding tumor deposit in the mesocolon or colic wall and dilation of the proximal transverse colon contributed to the evolution of the fistula proximal to the main tumor mass.

Cross-sectional imaging is the cornerstone of diagnosis and defines the anatomy of a suspected gastro- or duodenocolic fistula. Differentiating a duodenocolic fistula from a gastrocolic fistula is important for planning surgery. Abdominal contrast-enhanced CT scans play a vital role in delineating the fistula while also assessing locoregional spread and metastatic disease.

Endoscopy, though performed in this patient, failed to demonstrate either the tumor or the fistula. Failure to identify the tumor early was due to its laying beyond the reach of the sigmoidoscope, and a colonoscopy was warranted. Failure to identify the fistula by gastroduodenoscopy may have been the result of an incomplete or inadequate study, or it may have been due to the fact that the tract opened up to discharge colonic contents only when there was a rise in intracolonic pressure. An oral contrast study, if done preoperatively, may not have demonstrated the fistula for the same reason.

Treatment of malignant duodenocolic fistulae depends on several factors, such as the extent of local invasion, distant metastasis, and the general condition and comorbidities of the patient. Preoperative correction of fluid and metabolic abnormalities is essential for improved outcomes [6]. Patients frequently present with significant weight loss and malnutrition due to poor intake as well as malabsorption due to bacterial overgrowth in the proximal bowel secondary to the fistula [8]. Preoperative total parenteral nutrition may be beneficial in some of these patients [9].

Surgery is either curative or palliative, depending on the stage of the disease. Examples of curative strategies are a right colonic resection including the fistula tract and duodenal wall with primary closure of the defect or the use of a jejunal drainage loop or patch [10, 11]. Although better survival rates have been reported when colectomy is combined with pancreaticoduodenectomy (Whipple procedure), attendant morbidity and mortality, especially in malnourished patients, should be taken into consideration [12].

The metastatic and locally advanced disease in our patient necessitated a palliative approach. Palliative surgery is indicated when there is extensive local infiltration with retroperitoneal extension, extension to the major vessels, and distant metastasis. An ileotransverse anastomosis combined with a gastrojejunostomy may be performed in selected patients with advanced disease to relieve the obstruction and defunction the fistula [13]. Bypassing the fistula also minimizes bacterial contamination of the upper gastrointestinal tract and associated malabsorption. Considering the extent of disease and the overall poor nutritional status of this patient, an end ileostomy was performed in preference over an ileocolic anastomosis. Although survival after palliative surgery is variable, it is usually less than 12 months [5].

## Conclusions

Malignant gastric or duodenocolic fistulae are uncommon complications of locally advanced colonic malignancies with direct invasion to the duodenum. Although the characteristic clinical presentation of feculent vomiting suggests the diagnosis, cross-sectional imaging is confirmative in addition to staging the disease. Management is guided by disease stage, nutritional status, and the general condition of the patient and ranges from extensive bowel resection including the fistula and palliative options. Adjuvant chemotherapy may be of benefit in selected patients following surgery.

## Abbreviations

CT: Computed tomographic
DJ: Duodenojejunal
FOLFOX: 5-fluorouracil, folinic acid, and oxaliplatin

## References

[1] Soulsby R, Leung E, Williams N. Malignant colo-duodenal fistula; case report and review of the literature. World J Surg Oncol. 2006;4:86. 10.1186/1477-7819-4-86 PMC1698919 17147825

[2] Keighley MRB, Williams NS. Surgery of the anus, rectum and colon. London: W.B. Saunders; 1993.

[3] Ferguson CM, Moncure AC. Benign duodenocolic fistula. Dis Colon Rectum. 1985;28(10):852–4. 10.1007/BF02555491 4053898

[4] Lopez M, Hreno A. Iatrogenic duodenocolic fistula. Can J Surg. 1986;29(6):439. 3779548

[5] Calmenson M, Black BM. Surgical management of carcinoma of the right portion of the colon with secondary involvement of the duodenum, including duodenocolic fistula; data on eight cases. Surgery. 1947;21(4):476–81. 20290629

[6] Guraya SY. Malignant duodeno-colic fistula: a rare complication of colorectal cancer. J Clin Diagn Res. 2015;9(11):L01. 10.7860/JCDR/2015/16429.6800 PMC4668473 26673482

[7] Vagholkar KR. Small intestinal fistula. Bombay Hosp J. 2001;43(4):590.

[8] Steer ML, Glotzer DJ. Colonic exclusion bypass principle: its use in the palliative treatment of malignant duodenocolic and gastrocolic fistulas. Arch Surg. 1980;115(1):87–9. 10.1001/archsurg.1980.01380010073017 6153141

[9] Iuchtman M, Zer M, Plavnick Y, Rabinson S. Malignant duodenocolic fistula: the role of extended surgery. J Clin Gastroenterol. 1993;16(1):22–5. 8421139

[10] Majeed TA, Gaurav A, Shilpa D, Preeti J, Sanjay S, Manisha S, *et al.* Malignant coloduodenal fistulas - review of literature and case report. Indian J Surg Oncol. 2011;2(3):205–9. 10.1007/s13193-011-0099-x PMC3272171 22942613

[11] Gallagher H. Extended right hemicolectomy the treatment of advanced carcinoma of the hepatic flexure and malignant duodenocolic fistula. Br J Surg. 1960;47(206):616–21. 10.1002/bjs.18004720606 13826080

[12] Izumi Y, Ueki T, Naritomi G, Akashi Y, Miyoshi A, Fukuda T. Malignant duodenocolic fistula: report of a case and considerations for operative management. Surg Today. 1993;23(10):920–5. 10.1007/BF00311373 7905304

[13] Zer M, Wolloch Y, Lombrozo R, Dintsman M. Palliative treatment of malignant duodenoenteric fistulas. World J Surg. 1980;4(1):131–4. 10.1007/BF02393113 6155744

